# Hypoxia induced ferritin light chain (FTL) promoted epithelia mesenchymal transition and chemoresistance of glioma

**DOI:** 10.1186/s13046-020-01641-8

**Published:** 2020-07-16

**Authors:** Junhui Liu, Lun Gao, Na Zhan, Pengfei Xu, Ji’an Yang, Fan’en Yuan, Yang Xu, Qiang Cai, Rongxin Geng, Qianxue Chen

**Affiliations:** 1 Department of Neurosurgery, Renmin Hospital of Wuhan University, No.238, jiefang Road, Wuchang District, Wuhan, 430060 Hubei Province China; 2 Central Laboratory, Renmin Hospital of Wuhan University, Wuhan, China; 3 Department of Pathology, Renmin Hospital of Wuhan University, Wuhan, China

**Keywords:** Ferritin light chain, Hypoxia, Epithelial mesenchymal transition, Chemoresistance, Glioblastoma, Prognosis

## Abstract

**Background:**

Hypoxia, a fundamental characteristic of glioma, is considered to promote tumor malignancy by inducing process of epithelial mesenchymal transition (EMT). Ferritin Light Chain (FTL) is one of the iron metabolism regulators and is overexpressed in glioma. However, relationship between hypoxia and FTL expression and its role in regulating EMT remains unclear.

**Methods:**

Immunohistochemistry (IHC), western blot and public datasets were used to evaluate FTL level in glioma. Wound healing, transwell assays, CCK8, annexin V staining assay were used to measure migration, invasion, proliferation and apoptosis of glioma cells in vitro. Interaction between HIF1A and FTL was assessed by luciferase reporter and Chromatin immunoprecipitation (ChIP) assays. Subcutaneous xenograft model was established to investigate in vivo growth.

**Results:**

FTL expression was enriched in high grade glioma (HGG) and its expression significantly associated with IDH1/2 wildtype and unfavorable prognosis of glioma patients. FTL expression positively correlated with HIF1A in glioma tissues and obviously increased in U87 and U251 cells under hypoxia in a time-dependent manner. Mechanistically, HIF-1α regulates FTL expression by directly binding to HRE-3 in FTL promoter region. Furthermore, we found that knockdown FTL dramatically repressed EMT and reduced migration and invasion of glioma by regulating AKT/GSK3β/ β-catenin signaling both in vitro and in vivo. Moreover, our study found downregulation FTL decreased the survival rate and increased the apoptosis of glioma cells treated with temozolomide (TMZ). FTL expression segregated glioma patients who were treated with TMZ or with high MGMT promoter methylation into survival groups in TCGA dataset. Patients with methylated MGMT who had high FTL expression presented similar prognosis with patients with unmethylated MGMT.

**Conclusion:**

Our study strongly suggested that hypoxia-inducible FTL was a regulator of EMT and acted not only as a prognostic marker but also a novel biomarker of response to TMZ in glioma.

## Background

Glioma originated from the neuroectoderm and accounts for approximately 81% of primary malignant brain tumors [1]. Glioma is graded I-IV according to the 2016 World Health Organization classification of central nervous system tumors. Glioblastoma is the most common and malignant subtype and the dismal 2-year and 5-year survival rate are < 40 and < 10%,respectively [2]. Low grade gliomas (WHO I-II) usually progress to higher grade and eventually have poor outcomes despite treating with standard care (surgical resection combined with postoperative radiotherapy and chemotherapy) [3]. Therefore, it is urgent to understand the key molecular mechanisms in the malignancy progression of glioma to develop more effective treatments.

Hypoxia is a common pathological feature in glioma. Increasing grade of gliomas correlate with an increase in absence of oxygen [4]. Chronic hypoxia often leads to necrosis in tumor tissues, which is one of the most distinct characteristics of glioblastoma. Hypoxia microenvironment promotes glioma cells aggressive phenotype by upregulating hypoxia-inducible factor (HIF) family [5–7].HIF1A is highly expressed in glioblastoma and significantly correlated with IDH1/2 mutation [8]. Moreover, previous studies have demonstrated that hypoxia environment could induce the epithelial-mesenchymal transition (EMT) during the progression of glioma by regulating several pathways, such as Wnt/β-catenin [9], transforming growth factor β (TGF-β) [10] and Sonic Hedgehog (SHH) pathway [11]. This phenotype transition facilitated glioma cells easier to infiltrate the adjacent brain tissues and more resistance to chemo/radiotherapy [12]. However, the specific mechanism underlying which hypoxia promoting glioma malignancy remains to be further illustrated.

Ferritin was consisted of 24 units of heavy chain (FTH) and light chain (FTL). FTL had been widely recognized as one of the iron metabolism regulators for a long time. While in recent years, a growing number of studies have revealed the close relationship between FTL and tumor malignancies [13–16]. It was revealed that FTL could be used as a biomarker to discriminate benign and malignant tumors, and to predict the prognosis of patients with tumors [17, 18]. Besides, FTL was found to be overexpressed in various malignant tumors, and played a crucial role in regulating malignancy progress of cancers [19, 20]. Recent studies revealed that FTL could be upregulated on the post-transcriptional level by hypoxic conditions. Alveolar macrophages had 2.5 folds content of FTL when cells were exposed to hypoxia. Similar results were also observed in another study conducted by Sammarco et. HEK 293 cells were cultured in 1% oxygen and the results showed that FTL and FTH were differentially upregulated [21, 22]. These results suggested that FTL might be regulated under the hypoxia environment. While the effect of hypoxic environment on FTL expression and its regulation in process of glioma malignancy have not been well investigated so far.

In this study, we found that FTL was higher in HGG than in Low grade glioma (LGG). High FTL expression closely associated with wildtype IDH 1/2 and poor prognosis. We showed, for the first time that FTL was a hypoxia-responsive gene that significantly elevated under hypoxia in a time-dependent manner in U87 and U251 cells. Further analysis revealed that HIF-1A regulates FTL expression by directly binding to HRE-3 in the FTL promoter region. Functionally, we showed that FTL induced the EMT and promoted migration, invasion and chemo-resistance of glioma both in vitro and in vivo. Mechanistically, oncogenic role of FTL was functioned by regulating AKT/GSK3β/ β-catenin signaling.

## Materials and methods

### Clinical samples

Glioma tissues were obtained from the department of neurosurgery in Renmin hospital of Wuhan University from July 2015 to July 2018.A total of 142 paraffin-embedded glioma tissue were used for immunohistochemical staining. For western blot,28 glioma frozen tissues (stored at − 80 °C) of different grades were evaluated. Details of clinical information for all patients was presented in Table S1. None patients received any chemo- or radiotherapy before surgery. All patients signed informed consents and this study received the approval of the Ethics Committee of Renmin Hospital of Wuhan University (approved number: 2012LKSZ (010) H).

### Immunohistochemical (IHC) staining

The paraformaldehyde-fixed paraffin tissue microarray that contained 142 glioma tissues was used. The microarray was incubated with a primary anti-FTL monoclonal antibody (Abcam, USA: Ab109019) overnight at 4 °C. Images were captured using an Olympus BX40 microscope (Tokyo, Japan). Two individuals were separately responsible for the assessment of the results. The result was primarily based on the strength of staining and the number of positive cells.10 high magnification fields were randomly selected for observation. Positive staining rate was scored as: 0 points for less than 5%, 1 point for 5–25%, 2 points for 26–50%, 3 points for 51–75% and 4 points for 75%. Besides, the classification of the strength of staining was followed: non-staining is 0 points, light yellow is 1 point, brown yellow is 2 points, and brown is 3 points. Finally, multiply the two scores to get the final score which would be graded into 4 grades: negative (0 point), weakly positive (1–4 points), positive(5–8 points) and strongly positive(9–12 points). Final score less than 5 was defined as low expression and IHC score 5–12 was considered as high expression.

### Immunofluorescence staining

Cells were fixed with 4% paraformaldehyde for 15 min and penetrated with 0.5% Triton X-100 (made in PBS) at room temperature for 10 min. Then slides were washed with PBS 3 times. 1%BSA was added dropwise on the slides at room temperature for 30 min. Then we added a sufficient amount of the diluted primary antibody and placed it in a wet box, incubate at 4 °C overnight. Incubation with secondary antibody (Antgene, Wuhan, China) was performed in a wet box at 37 °C for 1 h under dark conditions. DAPI (ANT046, Antgene) was added in the dark for 5 min. Finally, slides were observed under a fluorescence microscope (Olympus BX51, Japan) to acquiring images.

### Cells, cell culture and transfection

Two human glioblastoma-derived cancer lines, U251 and U87 were purchased from the Cell Bank Type Culture Collection of the Chinese Academy of Sciences (Shanghai, China). Cell lines were identified by Procell Life Science&Technology Co.,Ltd. (Wuhan, China). Cell lines were all cultured at 37 °C under a humidified atmosphere of 5% CO_2_ by using Dulbecco’s modified Eagle’s medium (DMEM) supplemented with 10% fetal bovine serum (FBS) (Gibco, Invitrogen, Carlsbad, CA, USA). All cell lines were cultured without antibiotics. Short hairpin RNA (shRNA) targeted FTL and a scramble shRNA were purchased from Genechem Co., Ltd. (Shanghai, China). The target sequences against human FTL(5′-GGCGA GTATCTCTTCGAAA-3′) and scrambled shRNA(5′-TTTCGAAGA GATACTCGCC-3′) were cloned into the GV248 lentiviral vector. U251 and U87 cells were transfected with Lentivirus for 72 h and treated with puromycin(4 μg/ml) for 7 days.The specific small interfering RNA (siRNA) for FTL (siG143101050 18–1-5),HIF1A(siG0811494537–1-5), CTNNB1(siB08220115 751-1-5), HIF2A (siG170217101514–1-5) and scramble siRNAs were purchased from RiboBio (Guangzhou, China). Cells were cultured in a 6-well plate and transfected with lip2000(Invitrogen, Carlsbad, CA, USA) following the instructions of the manufacturer. After 48 h of transfection, the cells were used for subsequent experiments. FTL overexpression plasmid and a blank pcDNA3.1 vector were constructed.2 × 10^5^ cells were cultured in a 6-well plate and transfected with 2 ng plasmid using lip3000(Invitrogen, Carlsbad, CA, USA) following the instructions of the manufacturer. The cells were harvested after transfected for 48 h for further experiments. For in vitro hypoxia experiments, cells were cultured in a consistent 1% O _2_ hypoxic condition. The hypoxia mimetic cobalt chloride (CoCl_2_) (Sigma, NO.232696) was dissolved in sterile PBS and the final concentration of CoCl_2_ in the medium was 200 uM. To monitor resistance to temozolomide (TMZ), the U87 and U251 cells were treated with TMZ (Selleck, NO.S1237) at various concentrations for 24 h.

### Bioinformatics analysis

To clarify the expression and prognostic role of FTL in gliomas, we used the Gliovis database (http://gliovis.bioinfo.cnio.es/) and the UCSC Xena platform (http://xena.ucsc.edu/). Normalized RSEM gene-level RNAseq and corresponding clinical data of The Cancer Genome Atlas (TCGA),Rembrandt and IVY dataset were downloaded from Gliovis. Specific information on postoperative treatments (chemo/ radiotherapy) of glioma patients was downloaded from UCSC Xena platform. Besides, normalized mRNA expression (mRNA-array_693, (batch 1)) and clinical data were downloaded from Chinese Glioma Genome Atlas (CGGA). Low grade glioma was defined as WHO grade I-II and High grade glioma was defined as WHO III-IV according to the 2016 World Health Organization classification of central nervous system tumors [23].

### RNA isolation and RT-PCR

Total RNA of U251 and U87 cell lines were extracted using TRIzol regent (Invitrogen). We used PrimeScript RT reagent kit with gDNA Eraser (Takara, Tokyo, Japan) to prepared for cDNA and real-time PCR was performed by using SYBR Green II Mixture (TaKaRa) according to the manufacturer’s protocol. GAPDH was used for normalization and the comparative Ct method (ΔΔCt) was used to evaluate mRNA expression. The specific primer pairs were as follows: GAPDH (internal control gene) primer (forward primer, 5′-ACAACTTTGGTATCGTGGA AGG-3′; reverse primer, 5′-GCCATCACGCCACAGTTTC-3′); FTL primer (forward primer, 5′-CAGCCTGGTCAATTTGTACCT-3′; reverse primer, 5′-GCCAATTCG CGGAAGAAGTG-3′).

### Western blot

U251 and U87 were lysed in a modified RIPA buffer **(NO.P0013B, Beyotime Biotechnology, China)** on ice for about 30 min, then centrifuged at 12,000 rpm for 15 min. For frozen glioma tissues, we added 1 ml of RIPA lysate per 100 mg of tissues. The concentration of the sample was quantitatively determined by BCA protein assay. The lysate was mixed with loading buffer after heated at 100 °C for 5mins.In brief, equal protein amount was loaded on 8–12% SDS-PAGE and then transferred to a nitrocellulose membrane. Next PVDF membrane was blocked in 5% non-fat milk for 1 h and incubated with primary antibody at 4 °C overnight. Secondary antibodies (Antgene,Wuhan,Chian,1:10000) were used to incubate the membrane in shade environment at room temperature for 1 h.The membranes were visualized with Odyssey (LI-COR biosciences, USA). Primary antibodies used were presented in Table S2.Western blot analysis were repeated three times.

### Wound healing and transwell assay

Cells were seeded in a 6-well plate and cultured for a certain time to reach a > 90% confluence. The sterile pipette tip was used to scratch a linear wound and serum free DMEM was added for further culturing. Wound healing images were captured using an inverted microscope (Olympus BX51, Japan) and ImageJ software was used to analyze relative area of wound closed. For transwell assay, appropriated glioma cells were seeded into the upper well (Corning, USA) precoated with Matrigel (R&D, USA). The lower chamber was filled with 600 μl of DMEM containing 10% FBS. Transwell chambers were placed in an incubator (37 °C,5% CO_2_) for 24 h. Cells in the upper chamber were fixed with 4% paraformaldehyde for 15mins, stained with 0.1% crystal violet for 15mins and counted under an inverted microscope (Olympus BX51, Japan). We randomly selected 6 fields to count the number of invading cells in each set of experiments. All assays were repeated 3 times.

### Clone formation and cell count kit-8(CCK8) assay

1000 glioma cells were counted and seeded in 6-well plates. Cells were cultured with DMEM supplemented with 10% FBS and then Temozolomide was used to treat cells for 24 h.Cells were continued to be cultured in complete medium for about 10 days. Clones that contained more than 50 cells were scored. The clone formation rate was defined as the number of scored clones divided by the total cells seeded. For CCK8 assay,3000 cells were resuspended in 100 μl DMEM supplemented with 10% FBS and then added to a 96 well plate. Various concentrations of temozolomide were added. Cell proliferation was investigated using CCK8(Dojindo Molecular Technologies, USA) according to the manufacturer’s instruction.

### Flow cytometric analysis

Cells were seeded in a 6-well plate and treated with temozolomide(400 μM) for 72 h. Annexin V-PE/7- ADD kit (Becton Dickinson, USA) were used to measure the apoptosis of glioma cells. All operations were carried out according to the manufacturer’s instruction. In briefly, cells were harvested and washed three times with PBS. Then cells were stained with Annexin V-PE/7- ADD for 10 min under dark conditions. The apoptosis of samples was measured by FACS Calibur flow cytometer (BD Biosciences, USA). Early apoptosis and late apoptosis were summed and the total apoptosis rate was calculated.

### TUNEL assay

In Situ Cell Death Detection Kit was used to detected DNA fragmentation in apoptotic cells in xenografts according to the manufacturer’s protocol (Roche). In short, sections were deparaffinised at 60 °C for 20 min on a heat block and then incubated in xylene (3 × 5 min). Tissue was then rehydrated by washing in graded alcohol, 3 min for each, after which they were rinsed in PBS three times. After treated with 0.1%Triton X-100 and Proteinase K, the sections were incubated with TUNEL reaction mixture and incubated with converter-POD. Subsequently, DAB was used to stain slides. An Olympus BX51 microscope (Olympus) was used for image acquisition.

### Luciferase assays

To investigate the role of interaction between HIF1A and FTL, we constructed FTL promoter-driven luciferase reporter plasmids and transfected into U87 and U251 cells. Cells were pretreated with si-HIF1A or scramble for 48 h and then exposed to 1% O_2_ for 24 h.To further determine the direct binding between HIF1A and HREs in FTL promoter, we constructed mutant luciferase plasmid with ablation of HREs on FTL promoter by changing 5′–GCGTG-3′ to 5′–GCTCT-3′ and then co-transfected with plasmid containing the Renilla luciferase gene. Firefly luciferase activities were normalized using Renilla luciferase. Besides, TCF/LEF luciferase reporter (No.11542ES03) assay was performed using the Dual Luciferase Reporter Gene Assay Kit (Yeasen Biotech Co., Ltd. Shanghai, China) according to the manufacturer’s protocol. All experiments were repeated three times.

### Chromatin immunoprecipitation (ChIP)

ChIP was performed to explore the potential binding between the promoter region of FTL and HIF1A in glioma cells. U87 cells were incubated under the hypoxic condition for 24 h.Antibody against HIF1A was purchased from cell signaling technology (Danvers, USA). Precipitated DNAs that contained FTL fragments were then amplified using quantitative PCR. The sequences of three primers used to detect HREs in FTL promoter were as follows:1# (F): 5′-CGCAGGGCTTCTCTTTGTGG-3′, (R): 5′- TGAACAGTGTCTCTGAAGTTGCC -3′; 2# (F): 5′- CCACAACGCAGGGCTTCT C-3′, (R): 5′-TTTGGAGACAACTCACAGACTTCG -3′; 3# (F): 5′- CGCAGG GCTTCTCTTTGTGG -3′, (R): 5′- GGAGTGGAAATGGGGAGGAATG − 3′.

### In vivo experiments

All nude mice were purchased from Shulaibao (Wuhan,China) Biotechnology Co., Ltd. Animal feeding and experimental operations were in line with the guidelines of the Animal Ethics Committee of Wuhan University People’s Hospital. Stably transfected U87 cells that were growing in the logarithmic phase were prepared. Cells were resuspended in PBS at a concentration of 5 × 10^6^ cells/100 μL and then subcutaneously injected into the armpits of 5-week-old Balb/c nude mice. For in vivo temozolomide (TMZ) treatment, Six nude mice were randomly divided into two groups. Starting on day 5, the mice were injected intraperitoneally with TMZ (50 mg/kg) for 5 days. After subcutaneous implantation, the condition of the nude mice was observed daily. Recorded dynamic changes in the size of subcutaneous xenografts (longest diameter * shortest diameter ^2^/ 2). All nude mice were sacrificed on the 29th day after transplantation and the tumors were weighted.

### Statistical analysis

Data were presented as mean values ± standard deviation (SD) from at least three experiments. Student’s t-test was used to analyze the differences between two groups. One-way analysis of variance (ANOVA) was used for the comparison among three or more groups and the student-Newman-Keuls (SNK) method was used for post-analysis. Patients were divided into high and low groups according to the 50% cutoff point of FTL expression and Kaplan–Meier survival analysis was used to analyzed significance between groups. Univariate and multivariates Cox regression analysis was assessed by SPSS.21 (IBM, New York) software. Graphs production were performed by GraphPad Prism 5.0 software (GraphPad Software, Inc., La Jolla, USA). A *p* value of less than 0.05 was considered as statistical significance.

## Results

### FTL is overexpressed and associates with prognosis in high grade glioma (HGG)

**Fig. 1:**
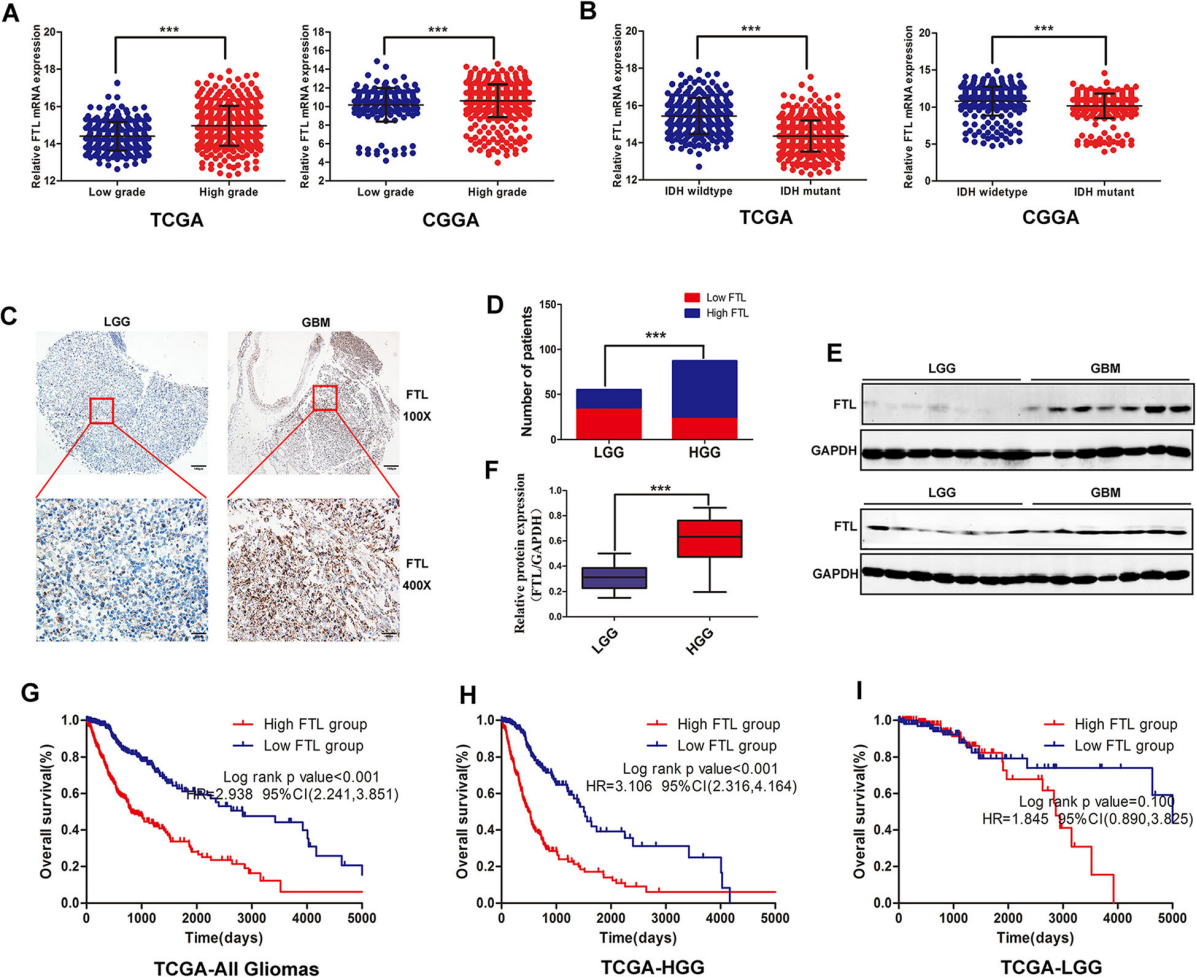
FTL is overexpressed and associates with prognosis in high grade glioma (HGG). **a** FTL mRNA expression in low grade glioma (LGG) and HGG in TCGA and CGGA datasets; **b** FTL mRNA expression in patients with wildtype and mutant IDH1/2 in TCGA and CGGA datasets;**c** Representative images of IHC staining of FTL in glioma tissues, GBM, glioblastoma;**d** Chi-square test was used for comparison between groups; **e** Western blot was performed to compared FTL expression in LGG and GBM, LGG, *n* = 14;GBM, n = 14,GAPDH used as loading control; **f** The images represented as the mean ± SD of three independent experiments.**g** Kaplan-Meier survival analysis for FTL expression in all glioma patients in TCGA and also analysis the prognostic role of FTL in HGG and LGG (**h** and **i**). The median value of FTL levels was set as the cut-off level. HR, hazard ration; CI, confidence interval. ***, P < 0.001, as compared to control

Our previous study revealed that FTL was elevated in glioma tissues when compared with non-tumor brain tissues by RT-PCR in a small glioma cohort [13]. While FTL expression in gliomas and its relationship with tumor malignancy remained poorly understood. In this study, we found that mRNA expression of FTL was enriched in HGG than in LGG using RNAseq data from three public datasets (Fig. 1a, Figure S1A). Moreover, FTL protein level was also elevated in HGG compared with that of LGG detected by IHC staining and WB (Fig. 1c-f). To explore the correlation between FTL and glioma aggressiveness, we compared FTL expression in different IDH1/2 status. FTL expression was significantly higher in IDH1/2 wildtype gliomas when compared with IDH1/2 mutant gliomas in TCGA and Rembrandt (Fig. 1b).

Then we tried to explore the prognostic value of FTL in gliomas by using public datasets. 50% cutoff point was used Kaplan-Meier analysis for FTL expression. Our results showed that glioma patients with higher FTL expression had worse overall survival than those with lower FTL expression in TCGA, CGGA and Rembrandt datasets (Fig. 1g, Figure S1B-C). Then we examined FTL expression on prognosis of HGG and LGG patients. The results showed that patients with high FTL expression had reduced survival time compared to patients with low FTL expression in HGG subgroup (Fig. 1h, Figure S1D-E). Though FTL expression segregated LGG patients into survival groups in CGGA and Rembrandt datasets (Figure S1F-G),FTL expression wasn’t significantly associated with survival time in TCGA dataset (Fig. 1i). Moreover, Cox regression analysis revealed that FTL was an independent risk factor for overall survival in patients with glioma (HR = 1.44,95%CI (1.06–1.95),*p* = 0.02,Table 1).

**Table 1:** Univariate analysis and multivariate COX analysis of clinical prognostic parameters in TCGA dataset

Variables	Univariate Cox regression		Multivariate Cox regression	
	HR(95%CI)	*P* value	HR(95%CI)	*P* value
Age (≥55y vs<55y)	5.27(3.95–7.03)	< 0.001	2.05(1.49–2.84)	< 0.001
Gender (Female vs male)	0.85(0.64–1.11)	0.23	–	–
WHO Grade (high vs low)	5.43(.68–8.04)	< 0.001	2.23(1.45–3.42)	< 0.001
IDH status (Wildtype vs mutant)	10.58(7.77–14.41)	< 0.001	5.17(3.54–7.55)	< 0.001
FTL expression (High vs Low)	2.87(2.15–3.83)	< 0.001	1.44(1.06–1.95)	0.02

TCGA, the cancer genome atlas;WHO,World Health Organization;IDH, Isocitrate dehydrogenase;HR,hazard ration

### Hypoxia induced FTL in a HIF-1α dependent manner

**Fig. 2:**
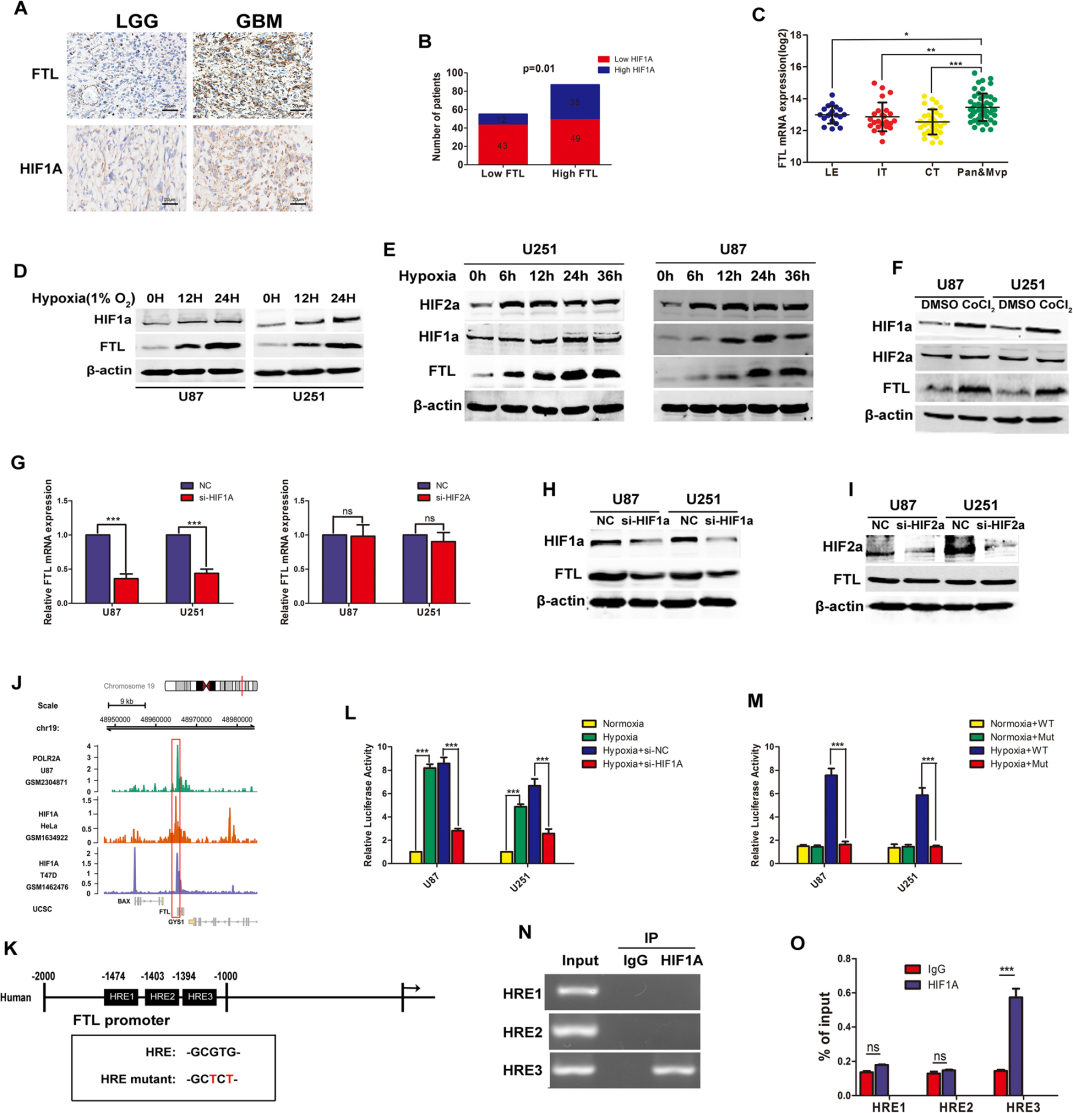
Hypoxia induced FTL in a HIF-1α dependent manner. **a** Representative images of IHC staining of FTL and HIF1A in glioma tissues and Chi-square test was used for comparison between groups **b**, Scale bars,20 μm; **c** Ivy glioblastoma atlas project (Ivy GAP) was used to analyzed FTL expression in different anatomic areas. LE, leading edge; IT, Infiltrating tumor; CT, cellular tumor; Pan, pseudopalisading cells around necrosis; Mvp, microvascular proliferation; **d** U87 and U251 cells were cultured under hypoxia and FTL expression was assessed by western blot. HIF1A was used as positive control.β-actin was used as loading control; **e** U251 and U87 cells were cultured under hypoxia. Whole cell lysates were collected and western blot was performed to find dynamic change of HIF1A,HIF2A and FTL protein expression.**f** Glioma cells were treated with CoCl_2_(400 mM) for 24 h,and HIF1A HIF2A, FTL expression were determined by Western blot.**g-i** Cells were transfected with siRNA-HIF1A or siRNA-HIF2A.mRNA and protein level of FTL,HIF1A and HIF2A were measured by RT-PCR and western blot, respectively. **j** Show for ChIP-seq profiles of HIF1A and HIF1A for a region of chromosome 19 obtained using RNAseq from Hela and T47D cells. Position of POLRA2A was used as positive control. **k** Putative hypoxia response elements (HREs) on the region of FTL promoter and graphic representation of mutant FTL promoter. **L** FTL promoter activity of U87 and U251 cells detected by Luciferase reporter assay in hypoxic or normoxic condition. Besides, **m** mutant-FTL-Luc or wildtype-FTL-Luc plasmids were then co-transfected with the Renilla luciferase reporter plasmid into glioma cells. Hypoxia maintenance time is 24 h. **n** and **o** Representative image of ChIP of HIF1A binding to FTL promoter under hypoxia. Anti-HIF1A and anti-IgG antibodies were used. All image represented as the mean ± SD of three independent experiments; *,P < 0.5;**,P < 0.01;***,P < 0.001, ns, no significance

Hypoxia condition is extremely common in glioma tissues and it is a crucial factor that contributes to the aggressive behavior of glioma. In our study, we tried to investigate the relationship between hypoxia and FTL expression in glioma. We used normalized RNAseq in TCGA datasets and found FTL expression significantly correlated multiply hypoxia-related markers, such as HIF2A, VEGFA, CA9 and PGK1(Figure S2A). Then the results of IHC staining showed that FTL was positively correlated with HIF1A in glioma tissues (Fig. 2a-b). Besides, the areas where FTL was highly expressed tended to co-expressed with higher expression of HIF1A (Figure S2B). Hypoxic area in glioma tissues always presented with more necrosis and microvascular proliferation (Mvp). Ivy glioblastoma atlas project (Ivy GAP) is a dataset that contains anatomic and genetic basis of glioblastoma at the cellular and molecular levels. FTL expression was higher in pseudopalisading cells around necrosis (Pan.) and Mvp areas than in other anatomic structures (Fig. 2c). These results indicated that FTL expression significantly associated with hypoxic environment. Then U87 and U251 cells were cultured under a hypoxic growth condition. We found that FTL expression was increased in U87 and U251 cells under hypoxia in a time-dependent manner (Fig. 2d).

To explore the role of hypoxia in regulating FTL expression, we again placed U87 and U251 cells under hypoxia condition. FTL protein level peaked at 24 h after hypoxia induction both in U87 and U25 cells, which was consistent with the pattern of increased HIF1A protein levels (Fig. 2e). However, HIF-2α expression was induced to the peak level at 6 h after hypoxia induction. To further determine the mechanism by which hypoxia induced FTL expression, we treated U87 and U251 cells with cobalt chloride (CoCl_2_,400 mM) for 24 h.The results showed that inhibition HIF1A degradation dramatically promoted the expression of FTL in glioma cells (Fig. 2f). Next, cells were transfected with siRNA-HIF1A or siRNA-HIF2A.The results of RT-PCR and Western blot data showed that knockdown HIF1A could reduce the FTL expression, while inhibiting HIF2A had no effect on FTL expression (Fig. 2g-i).IF staining also confirmed that knockdown HIF1A in glioma cells decreased FTL expression (Figure S2C).

**Fig. 3:**
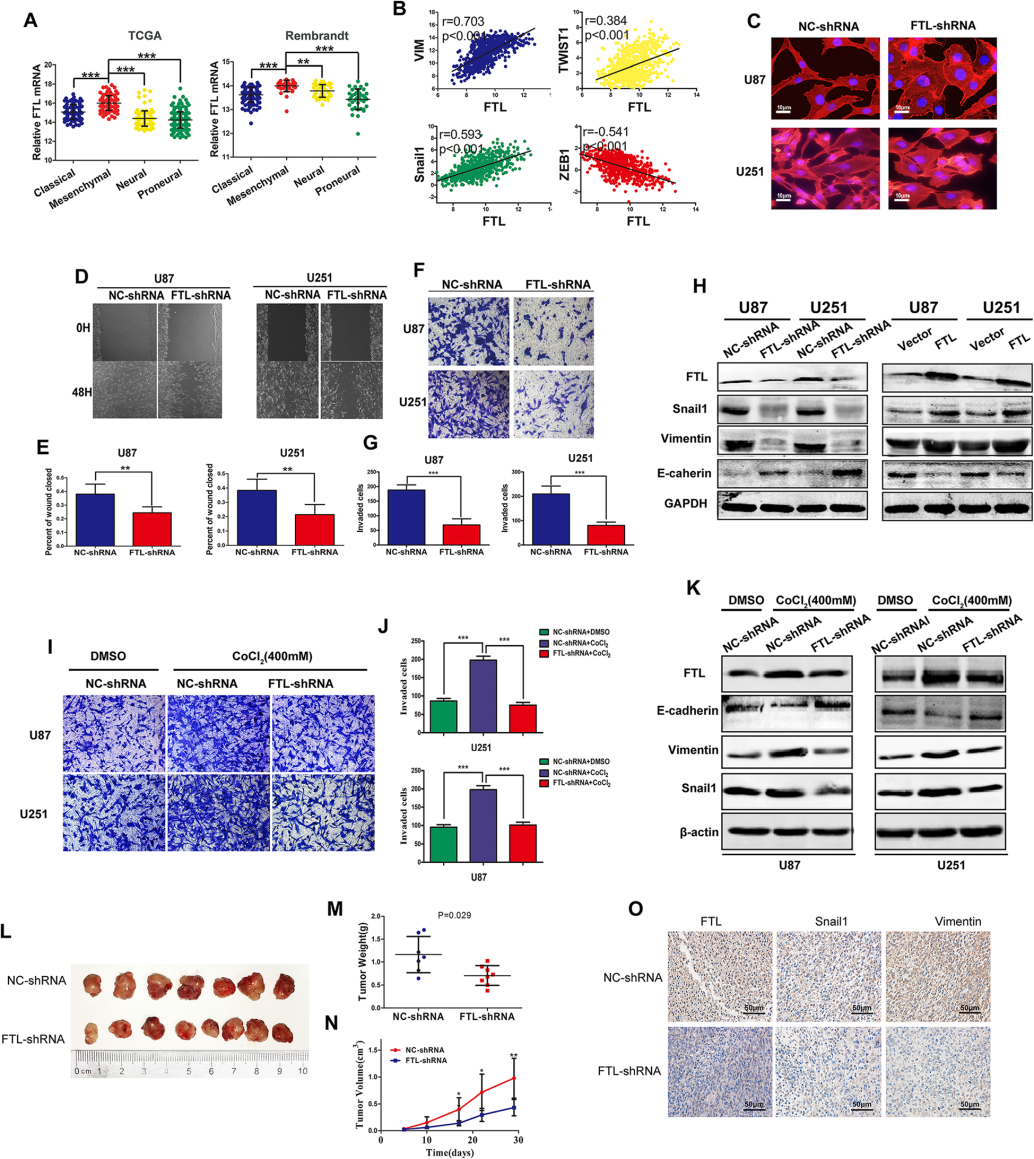
FTL promoted glioma cells epithelia-mesenchymal transition in vitro and in vivo. **a** Distribution of FTL expression in different glioma molecular subtypes based on RNAseq in TCGA and Rembrandt datasets. **b** Correlation between FTL with Vimentin, twist1, snail1 and ZEB1 in TCGA. Pearson test was used for correlation analysis. **c** Representative images of morphology of U87 and U251 cells transfected with NC-shRNA or FTL-shRNA. Cells were staining with phalloidin. Scale bars,10 μm. Wound healing (**d-e**) and transwell assays (**f-g**) were carried out to detect migration and invasion of glioma cells transfected with NC-shRNA or FTL-shRNA. Wound close percentage was calculated by Image J software (Rawak Software, Inc. Germany).*p*-values were determined by Student’s t-test. n = three independent experiments. **h** Western blot was used to detect expression of snail1,viementin and E-cadherin expression after knocking down or overexpression of FTL in U87 and U251 cells. GAPDH was used as loading control. **i-j** U87 and U251 cells were treated with DMSO or CoCl_2_(400 mM) for 24 h after transfected with NC-shRNA or FTL-shRNA.Transell assay for invasion detection and (**k**) western blot for E-cadherin,vimentin,snial1 expression measurement.β-actin was used as loading control. **l** Fifteen Nude mice were randomly divided into two groups. Images of the xenograft tumors formed in nude mice injected with FTL-shRNA cells and control cells. Tumor volume and tumor weight were calculated (**m-n**). **o** Representative images of IHC staining of FTL,snail1 and vimentin. Scale bars,50 μm. All images represented as the mean ± SD of three independent experiments;*,P < 0.5;**,P < 0.01;***, P < 0.001

In the promoter region of FTL, we found there were three hypoxia response elements (HREs),and bioinformatics prediction showed that promoter region of FTL may contain HIF1A binding site (Fig. 2j-k). We performed luciferase and ChIP assays to investigate whether HIF1A directly bond to FTL promoter. A full length wildtype FTL luciferase promoter reporter plasmids (FTL-Luc) was constructed. FTL promoter activity was increased in response to hypoxia and knockdown HIF1A in U87 and U251 cells inhibited hypoxia induced FTL promoter activity (Fig. 2l). Then we constructed mutant luciferase plasmid with ablation of HREs on FTL promoter by changing 5′–GCGTG-3′ to 5′–GCTCT-3′.Our results showed that transfection of mutant-FTL-luc in U87 and U251 cells could dramatically abrogate hypoxia-mediated FTL induction when compared with cells that were transfected with wildtype FTL-Luc (Fig. 2m).ChIP assay was then performed to further verified direct binding between HIF1A and FTL promoter. In chromatin fractions that were pulled down by anti-HIF1A antibody, RT-PCR data showed that only HRE-3 was detected (Figs. 2j, 3n-o).

### FTL promoted glioma cells epithelia-mesenchymal transition in vitro and in vivo

In order to explore the role of FTL in inducing EMT process, we analyzed glioma subtype-specific FTL expression in TCGA and Rembrandt datasets. FTL expression was extremely high in mesenchymal subtype compared with other subtypes (Fig. 3a). Besides, the area Under Curve (AUC) of FTL in predicting mesenchymal subtype of gliomas was 0.908 and 0.845 in TCGA and Rembrandt, respectively (Figure S3A-B). Moreover, we showed that FTL expression positively correlated with multiply EMT-related markers, such as Snail1,Vimentin and Twist1,but negatively correlated with ZEB1(Fig. 3b).

Next, we established stable FTL knockdown glioma cell lines using shRNA. Knockdown FTL obviously altered the morphology of U87 and U251 cells, namely from spindle shape to round shield with less pseudopods (Fig. 3c). Knocking down FTL inhibited the migration and invasion of U87 and U251 cells (Fig. 3d-g). Conversely, overexpression FTL significantly enhanced the migration and invasion of glioma cells (Figure S3C-F). Results of western blot showed that knockdown FTL significantly reduced the expression of vimentin and snail1, but increased E-cadherin expression (Fig. 3g). While overexpression FTL enhanced the expression of EMT markers in U87 and U251 cells (Fig. 3h). Immunofluorescence (IF) staining was then performed to verified our findings and the results showed knockdown FTL obviously altered expression of EMT related proteins of vimentin, snail1 in glioma cells (Figure S3G).

Since hypoxia increased FTL level in glioma, we explored whether FTL mediated hypoxia induced EMT of glioma cells. We used cobalt chloride, a HIF1A inhibitor, to mimic hypoxic conditions. Inhibition of HIF1A obviously enhanced the invasion of glioma cells, while hypoxia-enhanced invasion of glioma cells was blocked by knocking down FTL expression (Fig. 3i-j).What’s more, silencing FTL significantly reduced the expression of hypoxia-enhanced EMT markers, such as snial1 and vimentin (Fig. 3k).

Finally, we performed in vivo experiments. Weight of tumors in FTL-shRNA group was dramatically decreased compared with tumors in NC-shRNA group(0.71 ± 0.08 vs 1.17 ± 0.15,*p* = 0.014, Fig. 3l-m). Also, the growth of xenografts in FTL-shRNA group were smaller than that of NC-shRNA group (Fig. 3n). Then we used IHC to detect expression of EMT markers and the results showed that inhibition of FTL significantly reduced expression of snail1 and vimentin in vivo (Fig. 3o).

### FTL promoted EMT through AKT/GSK3β/β-catenin signaling

**Fig. 4:**
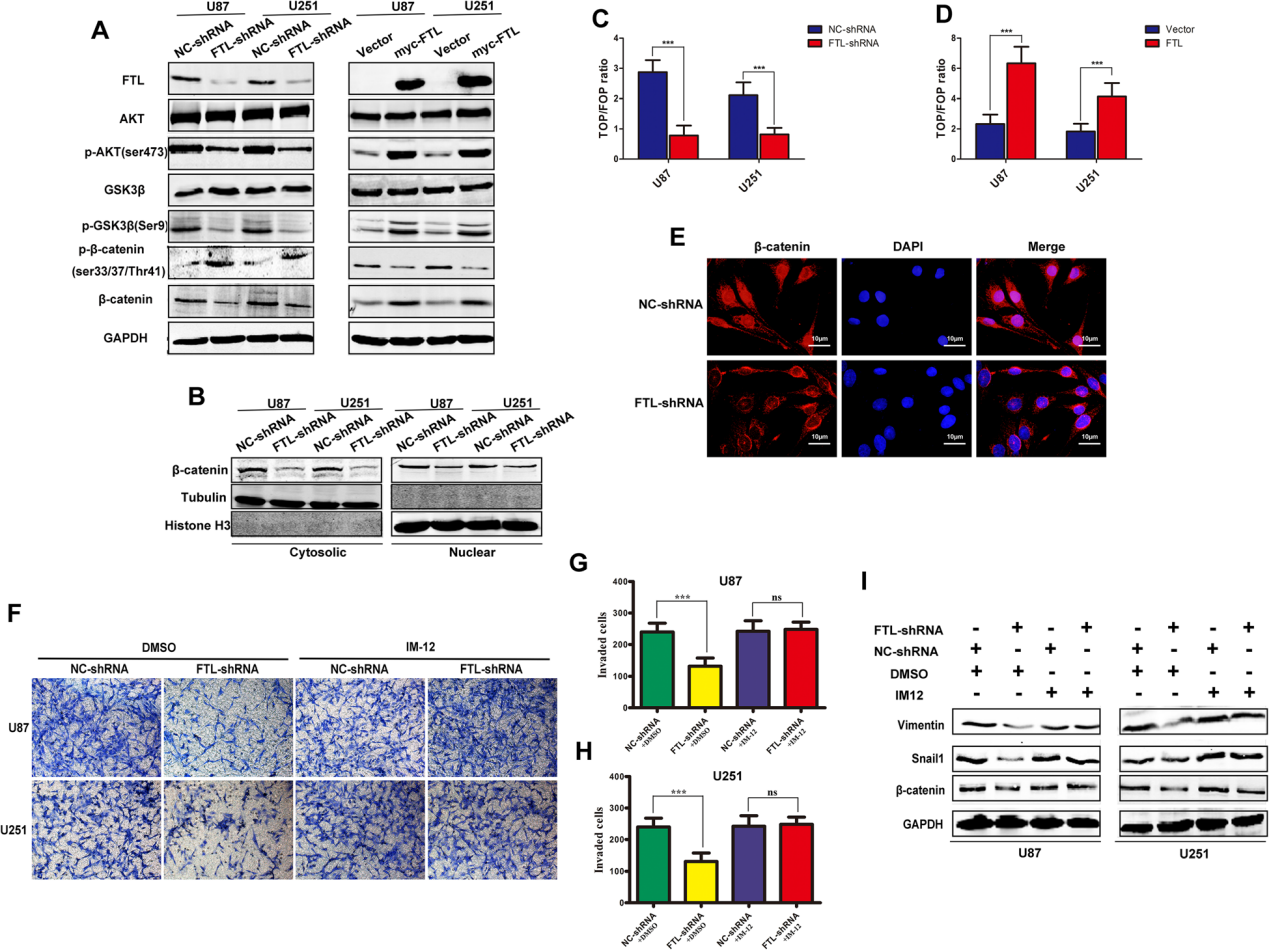
FTL promoted EMT through AKT/GSK3β/β-catenin signaling. **a** Western blotting analysis of AKT, p-AKT (ser473),GSK3β,p-GSK3β(ser9), β-catenin and p-β-catenin was performed in FTL silenced or FTL forced U87 and U251 cells. GAPDH was used as loading control. **b** Cytoplasmic and nuclear fractions of U87and U251 cells were extracted separately, and then expression of β-catenin was detected by western blot. Tubulin as cytoplasmic loading control and Histone H3 as nuclear loading control. **c-d** Next, TCF/LEF luciferase reporter assay was used to detect activity of β-catenin signaling in FTL-shRNA or FTL-plasmid transfected glioma cells. ***, *P* < 0.001. **e** Immunefluorescence staining confirmed that the distribution of β-catenin inside the cell. DAPI was used for nuclear staining; Scale bars,10 μm. **f-h** U87 and U251 cells transfected with NC-shRNA or FTL-shRNA were treated with DMSO or IM-12(GSK3β inhibitor) for 24 h.Transwell assay was used for invasion detection. **g** Western blot was employed for measurement expression of vimentin,snail1 and β-catenin. Three independent experiments were performed. *,P < 0.5;**, P < 0.01;***, P < 0.001

It is widely recognized that β-catenin signaling activation induced the expression of multiple EMT markers, such as Snail1,Twist1 and ZEB1 [24]. Our results showed that FTL positively correlated with β-catenin expression in TCGA (Figure S4A). In our validation cohort, we performed IHC staining and we found patients in high FTL expression group had higher level of β-catenin when compared with patients in lower FTL expression group (Figure S4B-C).What’s more, in glioma tissues with high FTL expression, the accumulation of β-catenin in the nucleus was more common than in low FTL expression glioma tissues. To further investigate the relationship between FTL and β-catenin, we performed in vitro experiments. We found that knockdown FTL reduced the expression of β-catenin, while overexpression FTL increase the expression of β-catenin (Fig. 4a). In our study, we found the cytoplasm and nucleus fraction of β-catenin were both decreased after repression of FTL by shRNA in glioma cells (Fig. 4b, e). Since nucleus part of β-catenin participated in WNT signal transduction, then we used luciferase reporter system to detect the activity of β-catenin signaling. Knockdown FTL reduced the TCF/LEF luciferase activity in U87 and U251 cells (Fig. 4c-d). These results indicated that β-catenin played crucial role in mediating FTL promoting EMT in glioma cells.

Then we performed WB to detect the expression of AKT,p-AKT,GSK3β, p-GSK3β and our results showed that knocking down FTL significantly inhibited the expression of p-AKT (ser473) and p-GSK3β(ser9),but the expression of total AKT and GSK3β had no significant change (Fig. 4a). While overexpression FTL in glioma cells increased p-AKT (ser473) and p-GSK3β(ser9). We found knockdown FTL in glioma cells increased the phosphorylation of β-catenin, while forced FTL expression decreased the phosphorylation of β-catenin (Fig. 4a). Furthermore, we selected a GSK3β inhibitor (IM-12) and β-catenin overexpression plasmid to complete rescue experiment. IM-12 is a novel GSK3β inhibitor which can increase the activity of β-catenin. We found that both IM-12 and β-catenin plasmid could reversed the effects of FTL knockdown on cell invasion in U87 and U251 cells (Fig. 4f-h, Figure S4D-F). Besides, they could both significantly abolished the effect of FTL on the expression of EMT markers, such as vimentin and snail1(Fig. 4i, Figure S4G). Taken together, these results indicated that FTL promoted EMT of glioma cells by regulating AKT/GSK3β/β-catenin signaling.

### FTL enhanced resistance to temozolomide (TMZ) chemotherapy in glioma cells

**Fig. 5:**
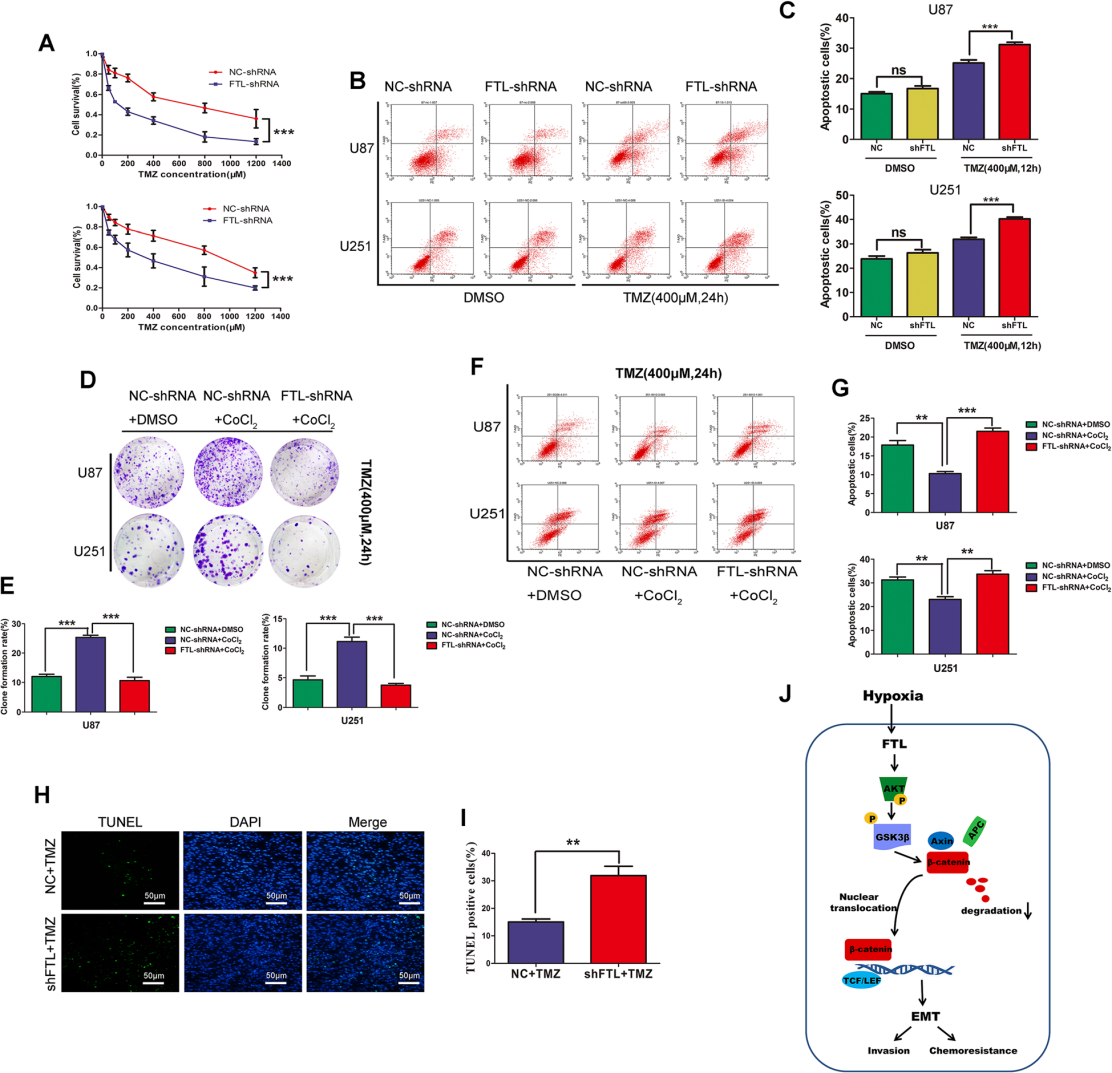
FTL enhanced resistance to temozolomide (TMZ) chemotherapy in glioma cells. **a** CCK-8 assay was used to detect the survival rate of U87 and U251 cells treated with different concentrations of TMZ. **b-c** Then U87 and U251 cells treated with certain TMZ concentrations(400 μM). The apoptosis cells were detected by flow cytometry and calculated by Fluorescence-activated cell-sorting (FACS). To investigate the role of FTL in mediating hypoxia-promoted EMT.U87 and U251 cells were treated with DMSO or CoCl_2_(400 mM) for 24 h after transfected with NC-shRNA or FTL-shRNA. Annexin V staining and clone formation were used to detect apoptosis (**f**-**g**) and proliferation (**d**-**e**) of glioma cells treated with TMZ(400 μM). Six Nude mice were randomly divided into two groups. Images of the xenograft tumors formed in nude mice injected with FTL-shRNA cells and control cells. All mice received two cycles of intraperitoneal injection of TMZ (50 mg/kg/day,5 day/cycle). **h**-**i** Representative images of TUNEL staining. Scale bars,20 μm. **j** Schematic showing hypoxia-induced FTL regulates EMT by regulating AKT/GSK3β/β-catenin signaling. All images represented as the mean ± SD of three independent experiments. *,P < 0.5;**, P < 0.01;***, P < 0.001

Accumulating evidence has shown that EMT phenotype is closely associated with chemotherapy resistance of glioma cells. First, we used the CCK-8 assay to detect the survival rate of U87 and U251 cells treated with different concentrations of TMZ and the results showed that inhibition of FTL enhanced the efficacy of TMZ and decreased cell survival rate (Fig. 5a). While overexpression of FTL dramatically increased the survival rate of glioma cells treated with different TMZ concentrations (Figure S5A-B). Moreover, inhibition of FTL increased apoptosis of U87 and U251 cells treated with certain TMZ concentrations(400 μM) detected by flow cytometry (Fig. 5b-c, while overexpression of FTL decreased the apoptosis of glioma cells when treated with TMZ concentrations (400 μM) (Figure S5C-E). Given these results, we assumed that FTL could enhanced TMZ resistance and decreased the cytotoxic effect of TMZ therapy on glioma cells. Moreover, we investigated whether hypoxia induced chemo-resistance was mediated by FTL Inhibition of FTL in glioma cells reduced the clone formation number induced by hypoxia (Fig. 5d-e). Besides, Knockdown FTL significantly induced higher apoptosis rate of cells under hypoxic condition compared with control (Fig. 5f-g). These results indicated that FTL downregulation could diminished, or partially eliminated hypoxia induced chemoresistance.

Next, we performed in vivo experiment. Six nude mice were randomly divided into two groups and received two cycles of intraperitoneal injection of TMZ (50 mg/kg/day,5 day/cycle).FTL stable knockdown U87 cells generated smaller tumors than the cells expressing NC-shRNA under TMZ treatment (Figure S5F). The growth of implanted tumors in mice injected with FTL-shRNA U87 cells were much lower than cells transfected with NC-shRNA (Figure S5G-H). Furthermore, results of IHC staining also revealed that knockdown FTL in U87 cell significantly reduced the MGMT expression and increased cleaved-caspase3 expression (Figure S5I). Our findings were verified by terminal deoxynucleotidyl transferase nick end labeling (TUNEL) and we found rate of TUNEL positive cells in FTL-shRNA groups was much higher than that of NC-shRNA group (Fig. 5h-i). These results strongly indicated that FTL enhanced resistance to TMZ chemotherapy in glioma and might be a promising target. Taken together, we demonstrated that hypoxia induced FTL promoted EMT by regulating AKT/GSK3β/β-catenin signaling, which subsequently enhanced invasion and chemoresistance of glioma cells (Fig. 5j).

### FTL is a novel biomarker of response to TMZ in glioma

**Fig. 6:**
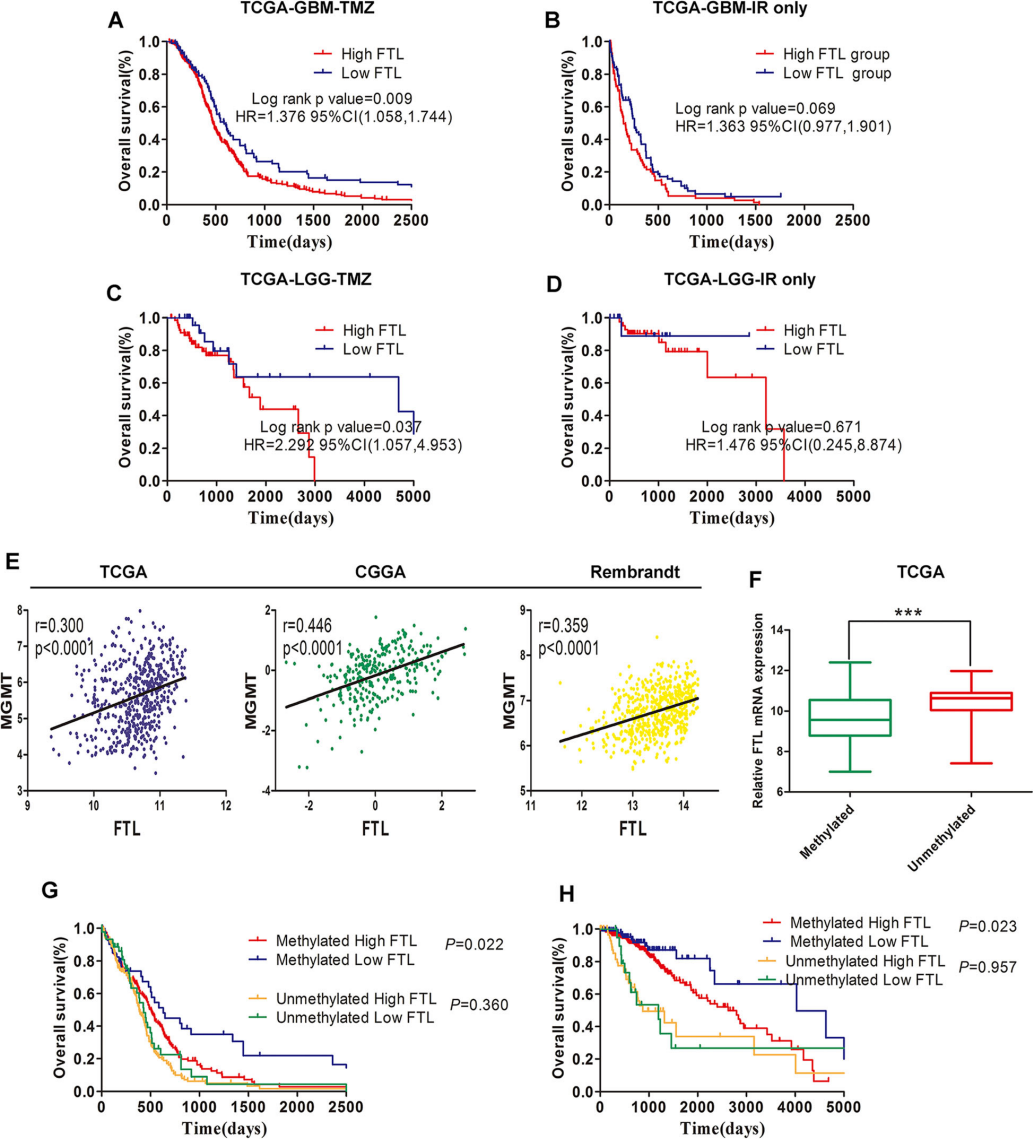
FTL is a novel biomarker of response to TMZ in glioma. **a-b** Kaplan-Meier overall survival curves in TCGA-GBM patients. Patient were divided into groups according to median FTL expression and treatment modality (TMZ at any time vs IR only). **c-d**TCGA-LGG patients were separated by median FTL expression and treatment modality (TMZ at any time vs IR only).**e** Correlation between FTL and MGMT in TCGA,CGGA and Rembrandt datasets. Pearson test was used for correlation analysis. **f** Relationship between FTL expression and MGMT status in TCGA-GBMLGG.**g-h** Patient were divided into groups according to median FTL expression and different methylation status of MGMT promoter in TCGA-GBM and TCGA-LGG. ***, P < 0.001

Since FTL mediated TMZ-resistance of glioma cells, we tried to investigate whether FTL could be used as a biomarker of TMZ therapeutic-response. We used TCGA datasets to analyze the effect of FTL expression on survival time of glioma patients with different treatments. We found that GBM patients with low FTL expression survived significantly longer than those with high expression if they were treated with TMZ at any time (Fig. 6a). While in patients who were treated with ionizing radiation (IR) alone, FTL expression did not segregate GBM patients into survival groups (Fig. 6b). Consistent with this findings, in LGG patients who expressed high FTL had shorter survival time than those expressed low FTL if they treated with TMZ at any time, whereas FTL expression wasn’t significantly associated with survival time of patients who were treated with IR alone (Fig. 6c-d). Considering the relationship between MGMT promoter methylation status and TMZ resistance, we explored the relationship between FTL and MGMT. First, we found that FTL expression was positively correlated with MGMT expression in glioma tissues in three public datasets (TCGA,CGGA and Rembrandt) (Fig. 6e). In TCGA, patients with unmethylated MGMT promoter had higher level of FTL than those with methylated MGMT promoter (Fig. 6f). These results showed that FTL might be a crucial gene involved in chemoresistance of glioma to TMZ.

Besides, FTL expression only segregate GBM patients into survival groups in tumors with high MGMT promoter methylation, while FTL expression was not informative in tumors with low MGMT promoter methylation (Fig. 6g). Furthermore, in LGG with high MGMT promoter methylation, FTL expression identified distinct survival groups. While FTL expression did not segregate LGG patients with low MGMT promoter methylation into survival groups (Fig. 6h). Our data showed that patients with methylated MGMT who had high FTL expression presented similar prognosis with patients with unmethylated MGMT. Taken together, these results suggested that FTL was a novel biomarker in predicting TMZ response in glioma patients.

## Discussion

Hypoxia environment is a fundamental characteristic of malignancy of glioma. The aggressive clinical behavior of glioma are closely related to the tension of oxygen in tumor microenvironment [25]. Hypoxia is considered to be a major driver of malignancy progression and treatment resistance of glioma. Hypoxia inducible factor 1(HIF1) had long been recognized as regulator of the mechanism of hypoxia-promoted progression in glioma [26].. Once cells are under hypoxic condition, HIF-1α will be gradually accumulated and then translocate to the nucleus. Subsequently, HIF-1α binds with HIF-1β to form a stable complex which can bind with the hypoxia responsive elements (HREs) in the promoter region of target gene [27]. The promoter of the ferritin gene contains regions of HREs that may interact with HIF-1α.Mitochondrial Ferritin (FtMt) is a form of ferritin distribution in mitochondria. FtMt was proved to be an potential target gene of HIF1a,as well as stabilized HIF1a in a hypoxic environment by binding to the HREs that located in the promoter regions of human FTMT gene [20]. In this study, we found a novel hypoxia response gene, FTL, which obviously increased under hypoxia in a time-dependent manner. Besides, there were three HREs in the region of FTL promoter and HIF-1α, not HIF2α regulates FTL expression by directly binding to HRE-3 in FTL promoter. FTL expression significantly correlated with glioma grade. As it was described before that increasing grade of gliomas correlates with an increase absence of oxygen [4]. So it was reasonable to infer that the high expression of FTL in HGG might be caused by the ubiquitous hypoxic-microenvironment in glioma.

Ferritinwas reported to play crucial role in regulating several solid tumors, including glioma [16, 28, 29]. Glioma cells were considered to undergo EMT during tumorigenesis and progression to higher grade. Recently, several studies uncover closely associated between ferritin and epithelial- mesenchymal transition (EMT). Previous study found that FTL was downregulated in osteosacrcoma (OS) and overexpression FTL in MG-63 cells enhanced the invasion and altered the expression of multiply EMT-related markers [30]. On the contrary, ferritin was evaluated by means of western blot in breast cancer cell lines, and results showed that FTL level was significantly elevated in mesenchymal phenotype cell lines compared with epithelial phenotype [17]. These findings indicate importance of FTL in process of EMT. However, the specific molecular mechanism of how FTL regulates EMT remains unclear. Moreover, authors of another study demonstrated that FTL was a marker of breast tumors with an aggressive phenotype [31]. Consistent with this finding, we found that FTL expression was mainly enriched in mesenchymal subtype and correlated with multiply EMT-related markers in TCGA and CGGA datasets. Also, knocking down FTL dramatically altered glioma cell to blunt morphology and reduced the migration and invasion of glioma cells, as well as alter expression of snail and E-cadherin. The reversal of the EMT process is accompanied by a decrease in the expression of EMT transcription factors and remarkable decline in cell invasiveness [31]. Our study provided solid evidence FTL might be a novel regulator of EMT in glioma. Targeting crucial control mechanisms of EMT could prevent the transformation from epithelial to mesenchymal subtype, which inhibited progression and enhance therapeutic effect of glioma [32].

Accumulating evidence shows that nuclear accumulation of β-catenin play crucial role in regulating EMT [33, 34]. Recently, Dong Xiao et al. found that SPHK2, a direct target of miR-708, triggered a cascade of signals leading to the activation of Akt pathway and the phosphorylation of GSK-3β and finally to the nuclear translocation of β-catenin to regulate EMT in glioma cells [35]. The nuclear accumulation of β-catenin correlated with WHO grades and cytoplasmic- nuclear β-catenin was an independent prognostic factors in glioma [36].FTL positively correlated with β-catenin and the nuclear accumulation of β-catenin was more common in glioma tissues with high FTL expression. Therefore it was reasonable to infer that FTL may have a certain relationship with β-catenin. We found that knocking down FTL in glioma cells dramatically reduced nucleus accumulation of β-catenin and dramatical decrease of activity of β-catenin signaling detected by Luciferase reporter system. The nuclear translocation of β-catenin bond with TCF/LEF transcription factors to induce the expression of vimentin and snail1,and subsequently activated the EMT process [37]. In our study, inhibition of FTL inactivated AKT by phosphorylation (ser473) and decrease of the phosphorylation level of GSK3β (ser9). Generally, inactive GSK3β, together with Axin, adenomatous polyposis coli (APC), casein kinase 1 (CK1) stabilized β-catenin which subsequently resulted in nucleus translocation of β-catenin. Therefore, we inferred that FTL was a regulator of AKT/GSK3β/ β-catenin signaling. Using IM-12 or CTNNB1 plasmid significant reversed the oncogenic function of FTL in mediating EMT in glioma, which strongly indicated that FTL promoted EMT by regulating AKT/GSK3β/ β-catenin signaling.

Interaction between hypoxia and EMT was mediated by multiply genes and pathways. HIF1A might directly induced the expression of TWIST which promoted EMT by interacting with Ring1B and EZH2. However, multiply genes might as crucial mediators by which hypoxia induced EMT. Previous study revealed that FAT1 positively correlated with multiply hypoxia related genes and it was a potent regulator of EMT both via or independent of HIF1a in glioblastoma [38]. We found that hypoxia enhanced the invasion of glioma cells, while inhibition FTL in glioma cells could mostly eliminate hypoxia-promoted invasion. Consistent with these findings, expressions of EMT-related markers were also obviously altered. Overall, our study revealed that HIF1A directly bond with HRE-3 in the region of FTL promoter to enhance its expression and FTL might act as a crucial gene that regulated EMT process of glioma. Targeting FTL in glioma cells could dramatically inhibit EMT induced by hypoxia, which indicated that FTL could be a potential target for therapy.

As a consequence of EMT process, glioma cells gradually become more invasive, and the adhesion between cells and cells is reduced, which makes it easier to infiltrate the adjacent brain tissues or escape from chemo/radiotherapy [39, 40]. In addition, EMT alter the stem cell characteristics of tumor cells and express more stem cell markers, which facilitates glioma resistance to chemotherapy and more likely to relapse. Hongbo Guo et al. found that miR-203 expressed low in imatinib-resistant GBM cells(U87AR,U251AR), and ectopic expression of miR-203 obviously reversed EMT by directly targeting SNAI2,which sensitized glioma cells to chemotherapy [41]. Also the results of another study revealed that miR-140 that targeted CTSB signaling suppressed the mesenchymal transition of GBM and enhanced TMZ cytotoxicity [42]. Previous studies demonstrated that FTL was participate in chemo-resistance of human breast cancer cells and colorectal cancer and inhibition of FTL induced sensitivity of cells to chemotherapy agents [20]. Consistent with these findings, we found thatknocking down FTL significantly inhibited the proliferation and increased apoptosis of glioma cells treated with TMZ(400 μM). Both in vitro and in vivo showed that cells transfected with sh-FTL were more sensitive to TMZ,which resulted in more apoptotic cells. Together, FTL could enhanced TMZ resistance and decreased the cytotoxic effect of TMZ therapy on glioma cells. The possible mechanistic explanations of FTL-mediated TMZ resistance are that FTL may be an important upstream regulatory protein in the process of MGMT methylation. Besides, HIF1A can activate autophagy. It’s possible that hypoxia induced FTL may also affect the autophagic degradation of proteins to affect the resistance of TMZ.

The significance of FTL expression was also demonstrated by its correlation with the clinical prognosis of glioma patients. Upregulation of FTL expression in glioma had been found in several studies, but the relationship of FTL expression and prognosis of glioma has not been well documented. Through bioinformatics analysis and in-house cohort validation, we have identified FTL as a novel biomarker of prognosis, as well as response to TMZ in glioma. Considering the correlation between FTL expression and IDH1/2 or subtypes, use of combination molecular analysis containing FTL might provide a more effective method for predicting prognosis of glioma. Moreover, FTL can be secreted into blood by glioma cells, detecting the level of FTL in plasma may predict the prognosis of glioma. This gives us a hint that FTL may become an important indicator in glioma liquid biopsy. Certainly, more clinical research is needed to clarify these issues regarding biomarker of plasma FTL in glioma.

## Conclusion

The present study showed that HIF1A directly bond with HRE-3 in the region of FTL promoter. FTL promoted EMT of glioma by regulating AKT/GSK3β/ β-catenin signaling, which subsequently enhanced invasion and chemoresistance of glioma cells. Thus, we concluded that hypoxia-inducible FTL was a regulator of EMT and acted not only as a prognostic marker but also a novel biomarker of response to TMZ in glioma.

## Supplementary information

**Additional file 1 Table S1.** Clinical information for all patients.

**Additional file 2 Table S2.** Details of primary antibodies. KPS, Karnofsky Performance Status; IDH, isocitrate dehydrogenase, FTL, Ferritin light chain.

**Additional file 3 Figure S1. Elevated FTL expression associated with high grade and predicts poor prognosis in glioma (A)** Level of FTL mRNA in low grade glioma (LGG) and high grade glioma (HGG) in Rembrandt dataset. Data was represented as the mean ± SD. ***, P < 0.001.(B-C) Effect of FTL expression on prognosis of all glioma, HGG and LGG patients in CGGA and Rembrandt dataset. The median value of the FTL levels was set as cut-off and Kaplan–Meier analysis was used.HR, hazard ration; CI, confidence interval.

**Additional file 4 Figure S2. Hypoxia induced FTL expression (A)** Correlation between FTL expression with expression of HIF2A, VEGFA, CA9 and PGK1 in TCGA. Pearson test was used for analysis. **(B)**Co-localization of HIF1A(green) and FTL (red) in glioma tissues was measured by immunofluorescence staining, DAPI was used for nuclear staining; Scale bars,100 μm; **(C)** Furthermore, immune-fluorescence staining was used to detect FTL expression (green) after knocking down HIF1A(red) in glioma cells. DAPI was used for nuclear staining; Scale bars,20 μm.

**Additional file 5 Figure S3. FTL promoted migration and invasion of glioma (A-B)** ROC curve was used to evaluate the predictive ability of FTL on mesenchymal molecular subtypes in TCGA and Rembrandt. AUC, area under curve; CI, confidence interval. **(C-D)** Wound healing was used to detect migration of U87 and U251 cells transfected with Vector or FTL plasmid. Wound close percentage was calculated by Image J software (Rawak Software, Inc. Germany). **(E-F)** Transwell assay was employed to detect invasion of glioma cells. **(G)** Immunefluorescence staining of snail1 and vimentin in si-NC or si-FTL transfected cells. DAPI was used for nuclear staining; Scale bars,20 μm. *, P < 0.5; **, P < 0.01; ***, P < 0.001.

**Additional file 6 Figure S4. Correlation between FTL and β-catenin in glioma tissues**. **(A)** Correlation between FTL and β-catenin mRNA expression in TCGA. Pearson test was used for correlation analysis. **(B-C)** Representative images of IHC staining of FTL and β-catenin in glioma tissues. Scale bars,50 μm. Red arrow pointed nuclear accumulation of β-catenin; Chi-square test was used for comparison between groups; **(D-F)** U87 and U251 cells transfected with NC-shRNA or FTL-shRNA were then co-transfected with vector or CTNNB1 plasmid. Transwell assay was used for invasion detection. (G). Western blot was employed for detecting expression of Vimentin, Snail1 and β-catenin. **, P < 0.01, ***, P < 0.001, ns, no significance.

**Additional file 7 Figure S5. FTL enhanced TMZ resistance of glioma**. **(A-B)** CCK-8 assay was used to detect the survival rate of vector or FTL transfected U87 and U251 cells treated with different concentrations of TMZ.(C-D)U87 and U251 cells treated with certain TMZ concentrations(400 μM).**(C-E)** The apoptosis cells were detected by flow cytometry and calculated by Fluorescence-activated cell-sorting (FACS). Besides, **(F)** Images of the xenograft tumors formed in nude mice injected with FTL-shRNA cells and control cells. All mice received intraperitoneal injection of TMZ (50 mg/kg/day,5 day/cycle). **(G-H)** Tumor volume and tumor weight were calculated. (I) Representative images IHC staining of FTL, MGMT and cleaved-caspased3 in xenograft tumor. Scale bars,50 μm. All images represented as the mean ± SD of three independent experiments. *, P < 0.5; **, P < 0.01; ***, P < 0.001.

## Data Availability

The data that support the findings of this study are available from the corresponding author upon reasonable request.

## Abbreviations

ChIP: Chromatin immunoprecipitation
EMT: Epithelia mesenchymal transition
FTL: Ferritin light chain
HGG: High grade glioma
HRE: Hypoxia response elements
HIF: Hypoxia-inducible factor
IHC: Immunochemistry
IDH1/2: Isocitrate dehydrogenase 1/2
LGG: Low grade glioma
TMZ: Temozolomide
WB: Western blot
