# Supplementary material for: Hypoxia induced ferritin light chain (FTL) promoted epithelia mesenchymal transition and chemoresistance of glioma
Source: J Exp Clin Cancer Res. 2020 Jul 16;39:137. doi: 10.1186/s13046-020-01641-8 (PMC7364815; doi:10.1186/s13046-020-01641-8)
Supplement: Supplementary file 1 — Additional file 1 Table S1. Clinical information for all patients. [file 13046_2020_1641_MOESM1_ESM.docx]

**Table S1 Clinical information for all patients**

| Variables | Low FTL (n=55) | High FTL (n=87) | P value |
| --- | --- | --- | --- |
| Age | 44.53±16.38 | 48.86±15.42 | 0.24 |
| Female | 24 | 40 | 0.78 |
| KPS |  |  | 0.62 |
| ≥80 | 38 | 57 |  |
| 50-70 | 15 | 19 |  |
| ＜50 | 2 | 1 |  |
| Grade |  |  | ＜0.001 |
| Ⅰ-Ⅱ | 33 | 22 |  |
| Ⅲ-Ⅳ | 22 | 65 |  |
| IDH1/2 status |  |  |  |
| Mutant | 9 | 6 | 0.19 |
| Wildtype | 12 | 23 |  |
| Unclear | 34 | 58 |  |

KPS, Karnofsky Performance Status; IDH, isocitrate dehydrogenase,FTL, Ferritin light chain
