# Supplementary material for: Hypoxia induced ferritin light chain (FTL) promoted epithelia mesenchymal transition and chemoresistance of glioma
Source: J Exp Clin Cancer Res. 2020 Jul 16;39:137. doi: 10.1186/s13046-020-01641-8 (PMC7364815; doi:10.1186/s13046-020-01641-8)
Supplement: Supplementary file 2 — Additional file 2 Table S2. Details of primary antibodies. KPS, Karnofsky Performance Status; IDH, isocitrate dehydrogenase, FTL, Ferritin light chain. [file 13046_2020_1641_MOESM2_ESM.docx]

**Table S2 Details of primary antibodies**

| Primary antibody | Lot number | Company |
| --- | --- | --- |
| FTL | ab69090 | Abcam |
| GAPDH | 60004-1-Ig | Proteintech |
| β-actin | [20536-1-AP](http://www.ptgcn.com/products/ACTB-Antibody-20536-1-AP.htm) | Proteintech |
| β-tubulin | GB11017B | Servicebio |
| Histone H3 | 17168-1-AP | Proteintech |
| β-catenin | #9562S | Cell Signaling Technology |
| AKT | #9272S | Cell Signaling Technology |
| p-AKT(ser473) | #9271S | Cell Signaling Technology |
| GSK3β | 22104-1-AP | Proteintech |
| p-GSK3β(ser9) | ab131097 | Abcam |
| p-β-catenin(Ser33/37/Thr41) | #9561T | Cell Signaling Technology |
| Vimentin | 10366-1-AP | Proteintech |
| Snail1 | 13099-1-AP | Proteintech |
| E-cadherin | ab231303 | abcam |
| HIF1A | #36169S | Cell Signaling Technology |
| HIF2A | 26422-1-AP | Proteintech |
| Caspase 3 | 19677-1-AP | Proteintech |
| Cleaved-caspase3 | ab32042 | Abcam |
| MGMT | sc-271154 | Santa cruz |
