# Supplementary figures and images for: Hypoxia induced ferritin light chain (FTL) promoted epithelia mesenchymal transition and chemoresistance of glioma
Source: J Exp Clin Cancer Res. 2020 Jul 16;39:137. doi: 10.1186/s13046-020-01641-8 (PMC7364815; doi:10.1186/s13046-020-01641-8)

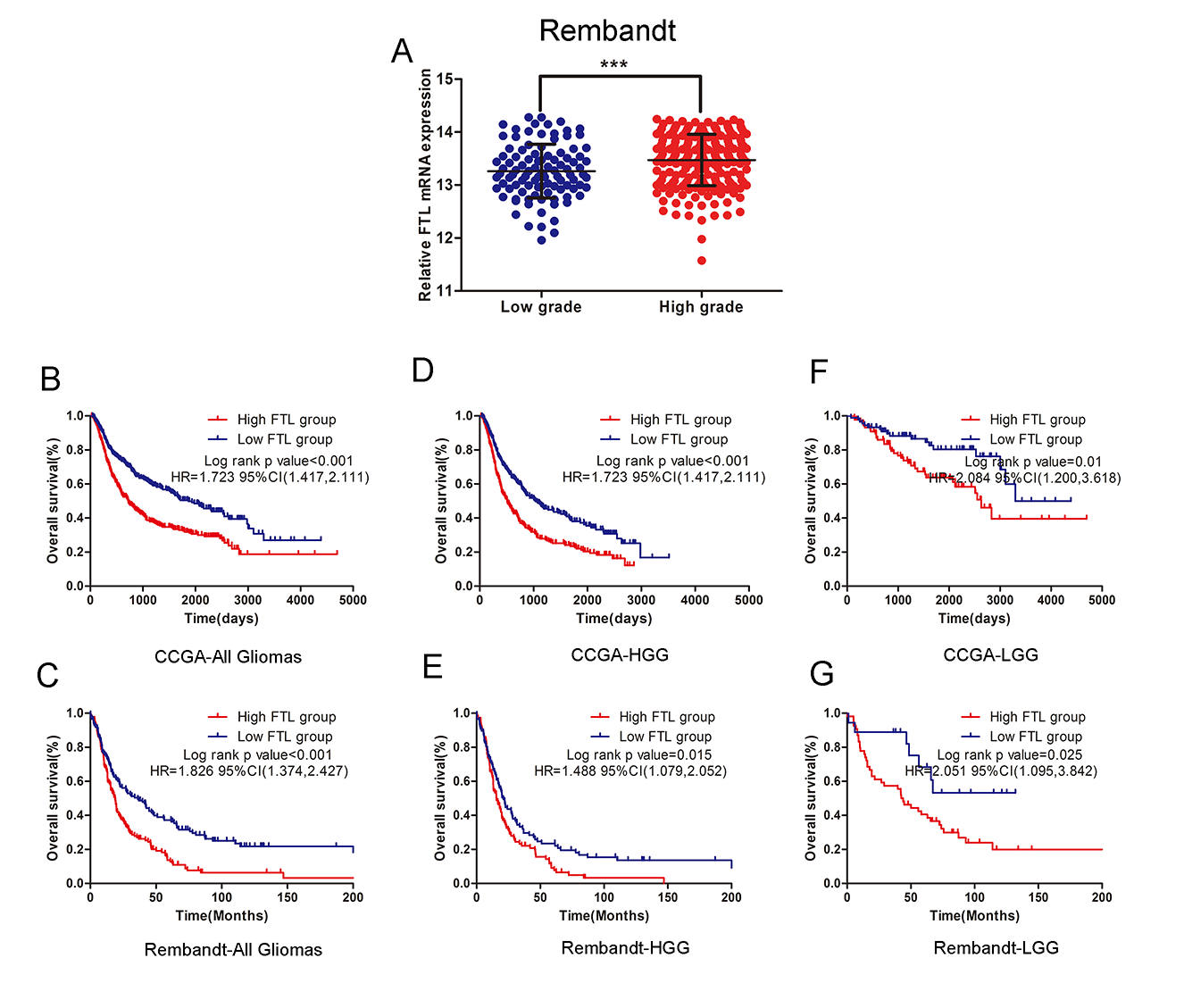

Supplement: Supplementary file 3 — Additional file 3 Figure S1. Elevated FTL expression associated with high grade and predicts poor prognosis in glioma (A) Level of FTL mRNA in low grade glioma (LGG) and high grade glioma (HGG) in Rembrandt dataset. Data was represented as the mean ± SD. ***, P < 0.001.(B-C) Effect of FTL expression on prognosis of all glioma, HGG and LGG patients in CGGA and Rembrandt dataset. The median value of the FTL levels was set as cut-off and Kaplan–Meier analysis was used.HR, hazard ration; CI, confidence interval. [file 13046_2020_1641_MOESM3_ESM.tif]

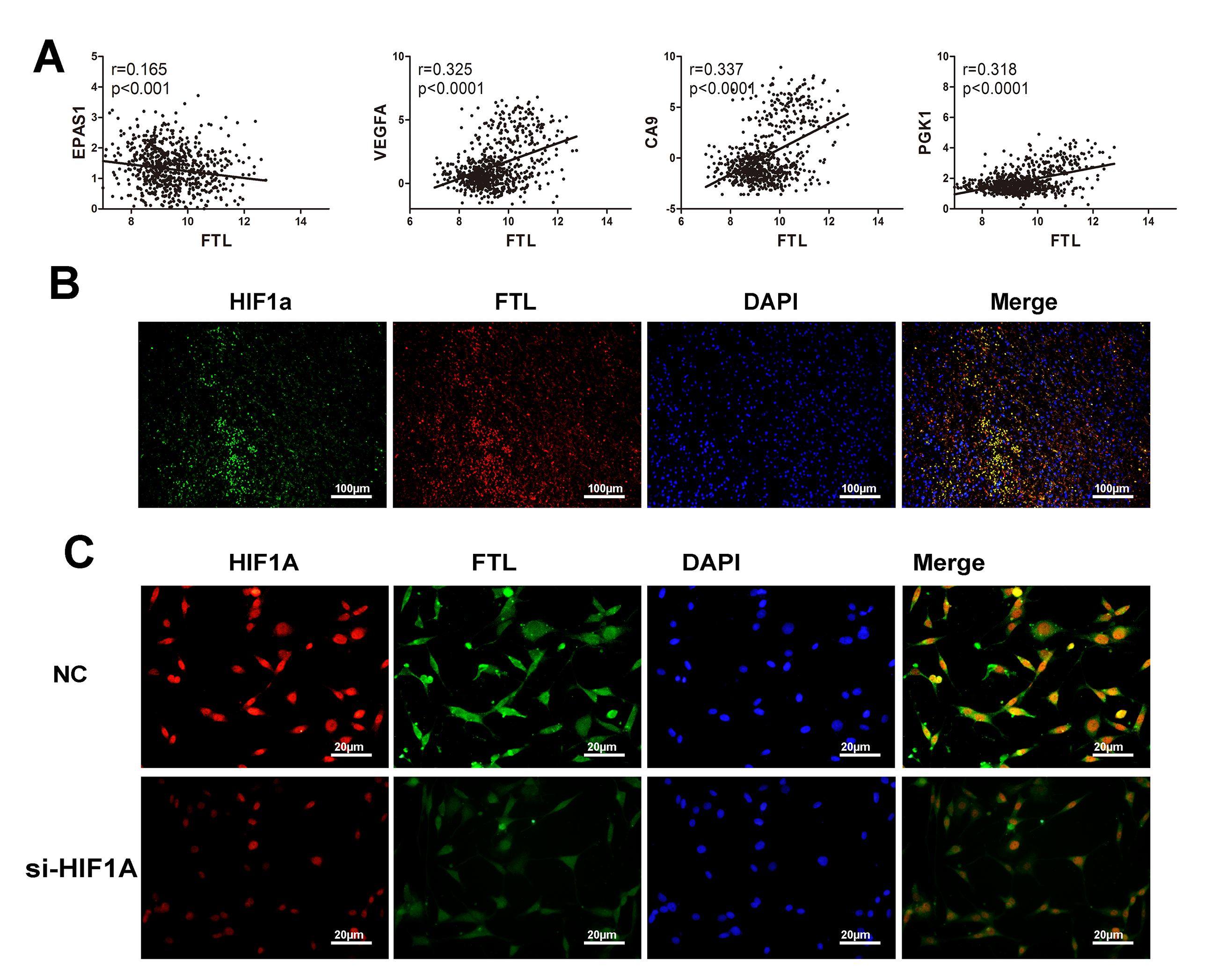

Supplement: Supplementary file 4 — Additional file 4 Figure S2. Hypoxia induced FTL expression (A) Correlation between FTL expression with expression of HIF2A, VEGFA, CA9 and PGK1 in TCGA. Pearson test was used for analysis. (B)Co-localization of HIF1A(green) and FTL (red) in glioma tissues was measured by immunofluorescence staining, DAPI was used for nuclear staining; Scale bars,100 μm; (C) Furthermore, immune-fluorescence staining was used to detect FTL expression (green) after knocking down HIF1A(red) in glioma cells. DAPI was used for nuclear staining; Scale bars,20 μm. [file 13046_2020_1641_MOESM4_ESM.tif]

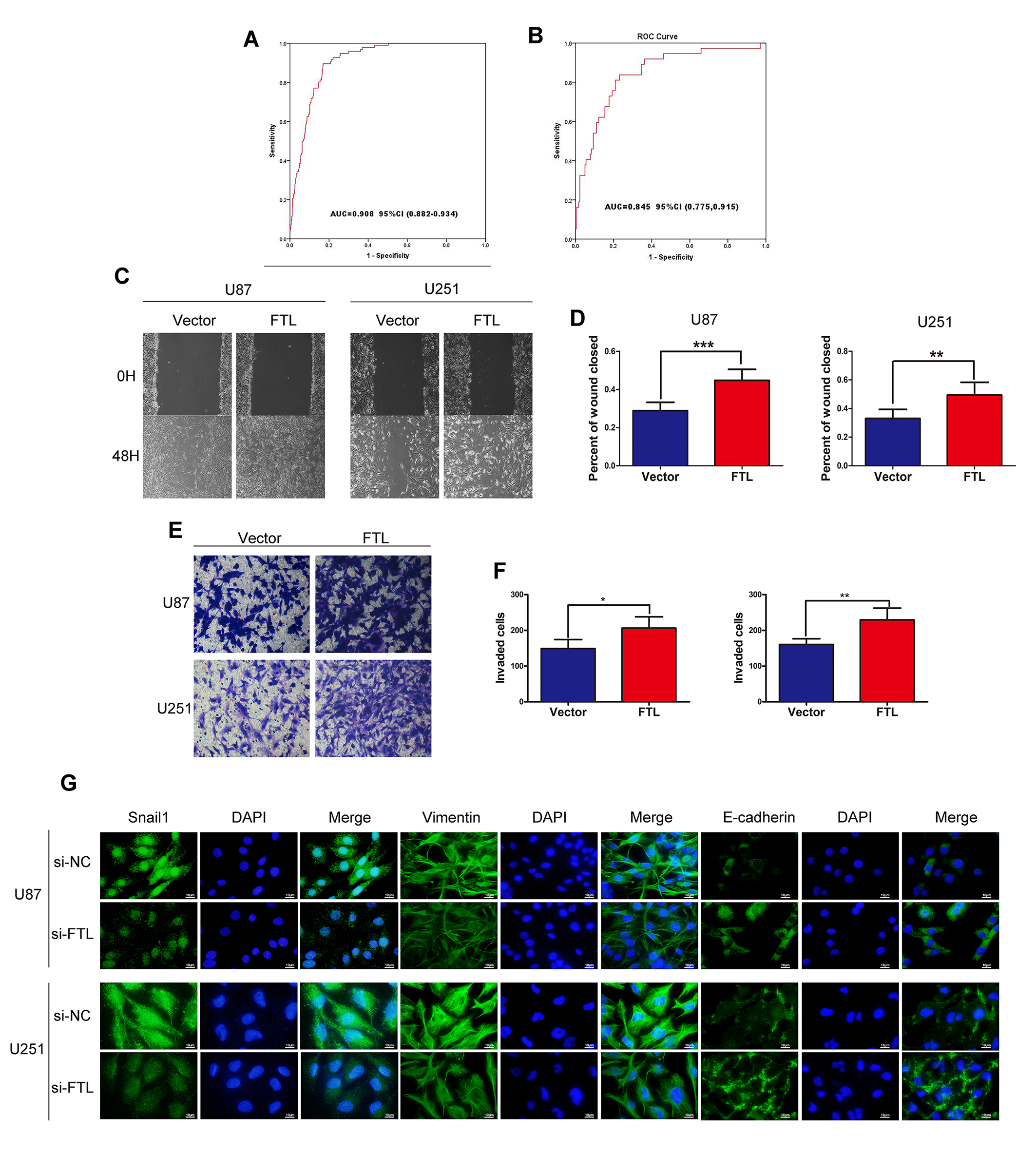

Supplement: Supplementary file 5 — Additional file 5 Figure S3. FTL promoted migration and invasion of glioma (A-B) ROC curve was used to evaluate the predictive ability of FTL on mesenchymal molecular subtypes in TCGA and Rembrandt. AUC, area under curve; CI, confidence interval. (C-D) Wound healing was used to detect migration of U87 and U251 cells transfected with Vector or FTL plasmid. Wound close percentage was calculated by Image J software (Rawak Software, Inc. Germany). (E-F) Transwell assay was employed to detect invasion of glioma cells. (G) Immunefluorescence staining of snail1 and vimentin in si-NC or si-FTL transfected cells. DAPI was used for nuclear staining; Scale bars,20 μm. *, P < 0.5; **, P < 0.01; ***, P < 0.001. [file 13046_2020_1641_MOESM5_ESM.tif]

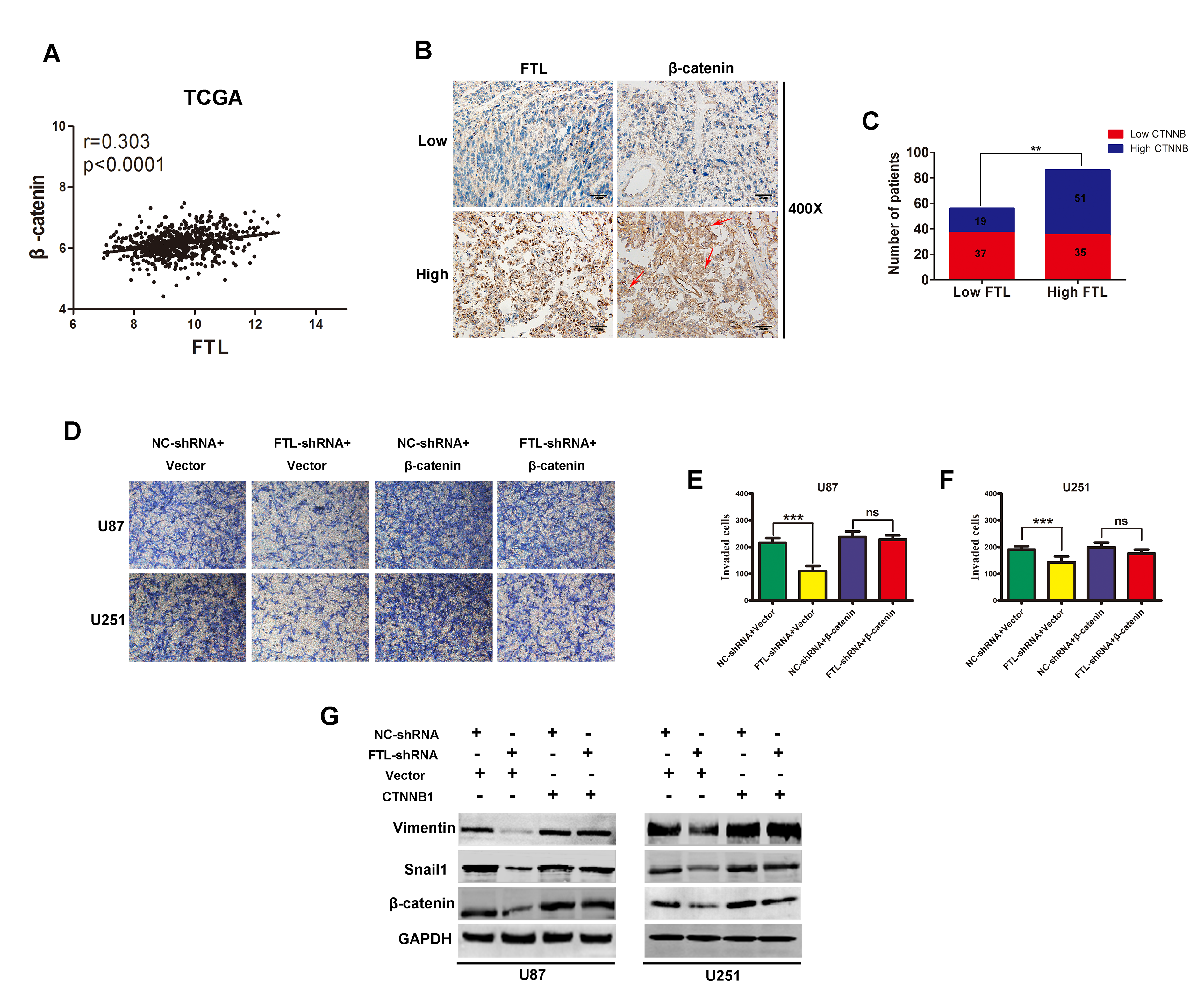

Supplement: Supplementary file 6 — Additional file 6 Figure S4. Correlation between FTL and β-catenin in glioma tissues. (A) Correlation between FTL and β-catenin mRNA expression in TCGA. Pearson test was used for correlation analysis. (B-C) Representative images of IHC staining of FTL and β-catenin in glioma tissues. Scale bars,50 μm. Red arrow pointed nuclear accumulation of β-catenin; Chi-square test was used for comparison between groups; (D-F) U87 and U251 cells transfected with NC-shRNA or FTL-shRNA were then co-transfected with vector or CTNNB1 plasmid. Transwell assay was used for invasion detection. (G). Western blot was employed for detecting expression of Vimentin, Snail1 and β-catenin. **, P < 0.01, ***, P < 0.001, ns, no significance. [file 13046_2020_1641_MOESM6_ESM.tif]

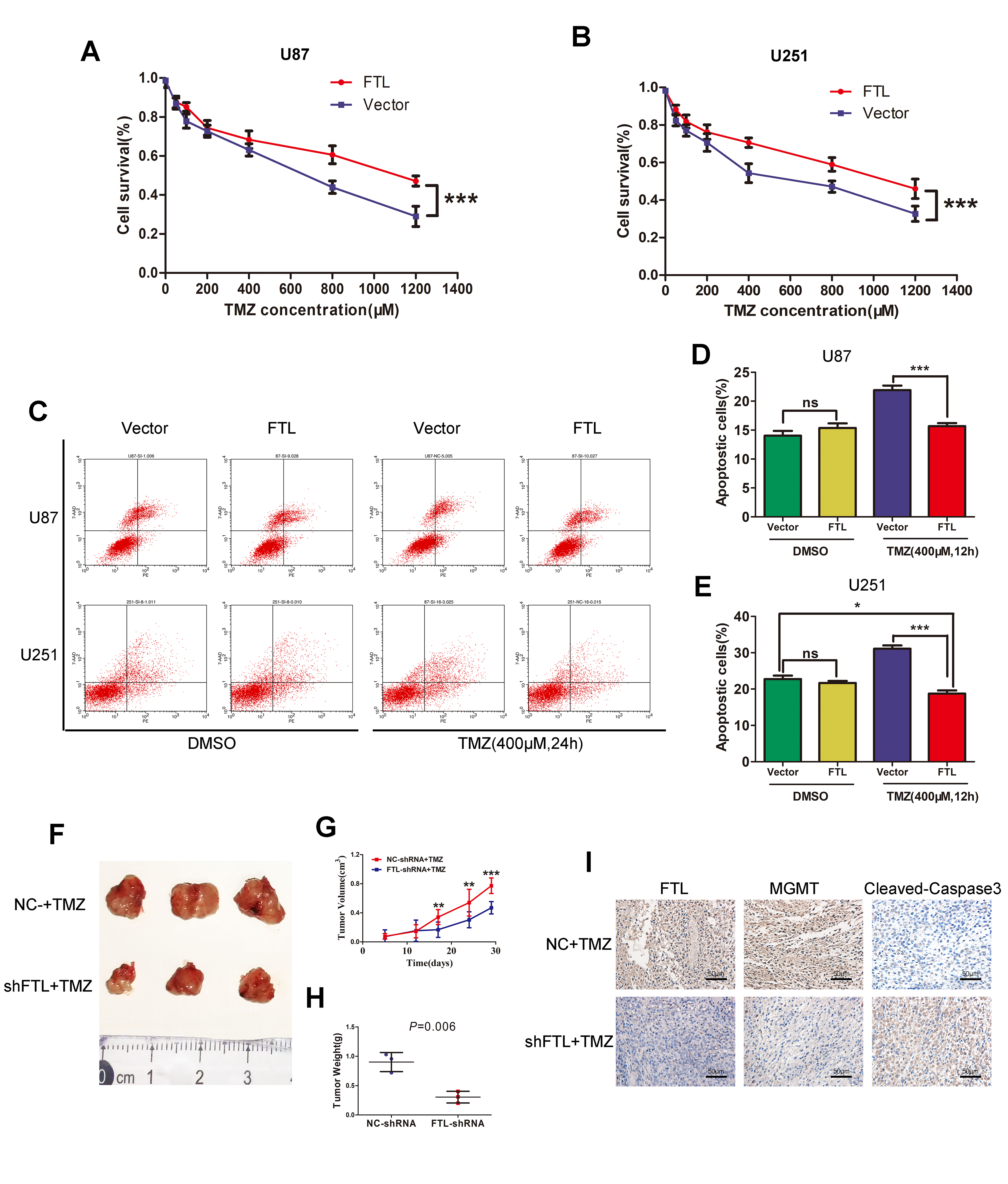

Supplement: Supplementary file 7 — Additional file 7 Figure S5. FTL enhanced TMZ resistance of glioma. (A-B) CCK-8 assay was used to detect the survival rate of vector or FTL transfected U87 and U251 cells treated with different concentrations of TMZ.(C-D)U87 and U251 cells treated with certain TMZ concentrations(400 μM).(C-E) The apoptosis cells were detected by flow cytometry and calculated by Fluorescence-activated cell-sorting (FACS). Besides, (F) Images of the xenograft tumors formed in nude mice injected with FTL-shRNA cells and control cells. All mice received intraperitoneal injection of TMZ (50 mg/kg/day,5 day/cycle). (G-H) Tumor volume and tumor weight were calculated. (I) Representative images IHC staining of FTL, MGMT and cleaved-caspased3 in xenograft tumor. Scale bars,50 μm. All images represented as the mean ± SD of three independent experiments. *, P < 0.5; **, P < 0.01; ***, P < 0.001. [file 13046_2020_1641_MOESM7_ESM.tif]
